# Exploring Repro‐Timing Harm and Benefit

**DOI:** 10.1111/bioe.70040

**Published:** 2025-10-27

**Authors:** Davide Battisti, Gary David O'Brien

**Affiliations:** ^1^ Interdepartmental Center for Research Ethics and Integrity National Research Council of Italy (CNR) Rome Italy; ^2^ Department of Philosophy and Hong Kong Catastrophic Risk Centre Lingnan University Hong Kong Hong Kong

**Keywords:** embryo cryopreservation, in vitro fertilization, person‐affecting views, procreative ethics, Population Ethics, repro‐timing harm and benefit

## Abstract

It is plausible that time of birth affects one's prospects for wellbeing. Being born during a war or recession might have a negative impact on early life and lifetime wellbeing. In natural reproduction, delaying conception does not result in the same child being born later, but rather a different child altogether; therefore, prospective parents cannot harm/benefit their children by choosing their time of birth. However, we argue that for prospective parents undergoing the IVF process, things are different. Since it is possible to freeze and store embryos indefinitely before implantation, parents can choose their child's time of birth. Because certain birth timings may better support wellbeing, this introduces the possibility of *repro‐timing harms and benefits*. This paper explores this new concept by outlining its theoretical assumptions and examining the moral reasons prospective parents in IVF might have for delaying implantation in the short, medium, and long term.

## 1 Introduction

The continuing development of assisted reproductive technologies increases parental control over the characteristics of their offspring. While this development increases procreative freedom, many authors believe that it also imposes novel moral obligations. Recent bioethical debate has focused on potential moral obligations grounded on procreative harms or benefits to specific individuals, particularly those which would be made possible by reproductive genome editing, a future and still hypothetical assisted reproductive technology that would enable us to modify in vitro embryos before implantation.

Our focus in this paper is on another kind of procreative harm and benefit made possible by existing reproductive technology. More specifically, one of the authors of this paper has recently argued that IVF and cryopreservation technology allow for a new category of person‐affecting harm and benefit—“repro‐timing harm and benefit” [1, 2]. These technologies allow prospective parents to choose the time of birth of their children without changing the identities of those children. Given that some times of birth offer better prospects for lifetime wellbeing than others, this raises the possibility that prospective parents engaged in the IVF process can benefit or harm their children by choosing their time of birth. Despite the fact that repro‐timing harm/benefit is already possible with existing technology, and – as we will argue – is less controversial than the harms/benefits of reproductive gene editing, it has remained almost entirely unexplored, particularly in its practical implications.

In this contribution, we intend to fill this gap, offering the first systematic discussion of this novel concept, first outlining its theoretical assumptions, and second examining its possible normative relevance in real‐world scenarios. The paper is structured as follows. In Section 2, we lay out the theoretical background that makes repro‐timing benefit/harm possible and outline the important distinction between person‐affecting and impersonal moral reasons. In Section 3, after briefly discussing some relevant insights from the debate on reproductive genome editing, we present repro‐timing harm and benefit. We point out that this concept is potentially less controversial than the concept of harm and benefit we encounter in reproductive genome editing. Moreover, we argue that repro‐timing harm and benefit helps reconcile our intuitions in reproductive ethics when prospective parents are concerned with the timing of reproduction. Having explained the theoretical background and demonstrated the possibility of repro‐timing harm/benefit, in the subsequent sections, we investigate whether there are any reasonably strong reasons for prospective parents engaged in the IVF process to delay implantation to avoid repro‐timing harms or secure repro‐timing benefits. We find that there are such reasons, and that they fall into three broad categories – short‐term reasons relating to the optimal times to be born within a given year, medium‐term reasons relating to prevailing social, economic, and personal circumstances, and longer‐term reasons relating to the possibility that scientific and cultural progress might dramatically improve life prospects for people born in future generations. We explore these reasons in turn in Sections 4, 5, and 6, respectively.

Throughout the paper, we assume that parents have strong (but not overwhelming) reasons to secure benefits for their future children, and that some degree of parental partiality is justifiable. For reasons of space, we mostly restrict our ethical analysis to the costs and benefits to parents and their future children and do not consider broader social or policy questions relating to repro‐timing harms and benefits. We conclude that prospective parents have strong pro tanto reasons to strategically implant embryos to secure repro‐timing benefits and avoid repro‐timing harms for their future children, especially in the short term, though we do not take a stance on whether they have all‐things‐considered reasons to do so.

## 2 Person‐Affecting Reasons in Reproduction

Discussions about procreative obligations in assisted reproduction depend on which kinds of reasons one considers relevant in the normative evaluation of reproduction. Consider the following case in the context of In Vitro Fertilization (IVF) and Preimplantation Genetic Diagnosis (PGD):

A couple decides to employ IVF and PGD to have a child and discovers that an embryo carries a mutation M, which will lead to a genetic disease that negatively impacts the future person P's well‐being or opportunities.

It is reasonable to ask whether the couple has a moral obligation to avoid selecting an embryo carrying mutation M. Authors who answer this question affirmatively generally believe that *impersonal reasons* matter morally, embracing the so‐called “impersonal view”. To understand this, we need to consider that the choice of one embryo over another not only determines the future individual's characteristics—whether or not the future child will have mutation M—but also their numerical identity. This assumes the “origin view” [3], according to which, a necessary condition for numerical identity is that each individual originates from a specific union of a particular sperm and a specific egg, and from no other.

If the selection of an embryo determines the identity of the future individual, this makes it difficult to justify a parental duty to avoid selecting an embryo with mutation M based on the potential *harm* to the future individual. The most widely accepted definition of harm is certainly the *counterfactual comparative* one [4, 5, 6, 7]: a person P is harmed by an event or condition X when X produces a state of affairs in which P is worse off compared to how they would have been if X had not occurred. Existence with M, however, is the only possible existence for P because if the parents had decided to select a different embryo instead, this person would never have existed. This means that we cannot compare two states in which P exists, one with condition M and one without it, since existence with M is the only possibility for P, and therefore, we cannot say that P has been made worse off. If this is so, then if the parents choose to implant the embryo with M, P has no complaint since they have not been harmed. But this conclusion may be difficult to accept. This puzzle is called Non‐Identity Problem [3].

Authors who embrace the Impersonal View believe that it is possible to solve the Non‐Identity Problem and explain the wrongness of the couple's choice without appealing to harm to an actual person, for instance, in terms of aggregate well‐being. This is the justification behind procreative principles like the Principle of Procreative Beneficence [8, 9], according to which, whenever IVF plus PGD is possible, reproducers have a significant moral reason to select the most advantaged child compared to the other possible children they could have.

Other authors, however, argue that it is questionable to rely on impersonal reasons and instead adhere to the *person‐affecting view*, according to which an action is right/wrong only if it benefits/harms an actual person—namely, a person whose identity does not depend on the action that harms or benefits them. From this perspective, the couple has done nothing morally wrong in selecting the embryo with M since the future child has not been harmed [7, 10, 11]. Since we want to explore concepts of harm and benefit in reproduction, our paper deals solely with reasons of this second kind.^1^ Furthermore, there are reasons to focus on a person‐affecting view. First, this may garner greater consensus, since, while some authors have denied the existence of impersonal reasons, person‐affecting reasons have never been denied. Secondly, other authors, while recognizing the existence of impersonal reasons, attribute greater significance to person‐affecting reasons, as seen in the so‐called Two‐Tier View [9, 14].

## 3 Repro‐Timing Harm and Benefit

The case of genetic selection discussed in the previous section does not exhaust the debate on procreative duties from a person‐affecting perspective. For instance, reasons of this kind have recently been extensively explored considering the recent development of genetic modification techniques such as CRISPR/Cas9, which, in the coming years, could lead to the clinical application of reproductive genome editing, through which it would be possible to modify the genome of embryos in vitro before uterine transfer, to prevent harmful traits or promote beneficial ones [15, 16]. Let us modify the previous example:

At the time of IVF employed by the couple, a genetic modification treatment is available that can safely, effectively, and economically remove M.

In this context, the future person P shares the same numerical identity with the embryo affected by mutation M. Therefore, existence with M is no longer the only possible scenario for P because there is a scenario in which P exists without M. By choosing to select the embryo with M without modifying it, the parents fail to avoid a harmful condition for P. In this case, unlike in the case of genetic selection, P would be born in a harmed condition that the parents could have avoided. In this regard, some authors argue that, considering the future availability of reproductive genome editing, parents would face new moral obligations toward their future children.

This idea has been extensively contested [17, 18, 19]. Here, we highlight two reasons for this controversy: first, it is controversial whether the embryo resulting from the modification is numerically identical to the embryo before the modification. In some contexts, genome editing might be seen not so much as a practice that harms or benefits the individual, but rather as one that creates a new individual. Call this the identity objection. A second reason relates to the fact that this technique remains hypothetical, as there is not yet any clinical application. Some have even argued that reproductive genome editing may never have any clinical utility [20, 21]. From this perspective, the harms hypothesized in the literature might never come to pass. Call this the applicability objection.

Addressing these issues is beyond the scope of this paper. However, they help us to introduce the concept of repro‐timing harm and benefit. As will become clearer in what follows, this new concept does not face either of the two challenges discussed above. Firstly, repro‐timing harm and benefit does not face the identity objection, since it does not require potentially identity‐changing modifications to an embryo. Secondly, repro‐timing harm and benefit avoids the applicability objection since it is possible with *current* assisted reproduction technologies, that is, IVF and cryopreservation of in vitro–produced embryos. This practice is often used to preserve more embryos than are transferred to the uterus during an IVF cycle, serving various purposes. For instance, if the first IVF cycle is unsuccessful, the couple can directly transfer cryopreserved embryos without the mother having to undergo ovarian stimulation and egg retrieval again. The couple or the individual may also choose to use these embryos to have a child at a later stage in life, when fertility may be lower. Additionally, this practice allows embryos to be used for research purposes. Cryopreservation has raised ethical issues, particularly regarding the fate of surplus embryos, which we will not explore in this paper. We simply acknowledge that cryopreservation enables embryos to be preserved for potential uterine transfer that could result in a live birth even years later. For example, in November 2022, a couple from Oregon gave birth to twins developed from embryos frozen over 30 years earlier, setting a record for the longest‐frozen embryos to result in a successful live birth [22].

Considering the possibility of cryopreserving embryos without compromising their characteristics and the possibility of their uterine transfer, prospective parents undergoing IVF could, in principle, choose to transfer the designated embryo or embryos at a different time than when they underwent the IVF process. If they create an embryo E at time t0 and the option to cryopreserve embryos and postpone pregnancy safely and effectively is available, there are at least two potential times in which E could be transferred into the uterus: t1 and t2. If life starting at t1 + 9 months can significantly undermine the well‐being of the future individual derived from E compared to starting at t2 + 9 months, then opting for t1 constitutes repro‐timing harm. A similar argument can be made for the repro‐timing benefit. Therefore, if parents have moral reasons to confer benefits and avoid inflicting harms on their children, the identification of repro‐timing harm or benefit may provide pro tanto reasons to delay implantation to give the child a better start in life.

Repro‐timing harm and benefit is in line with the well‐established attitude of many prospective parents who plan to have a child and wonder about the proper time to reproduce. For example, many people try to become more financially stable to ensure a better start for the child they will have. These considerations do not appear to be confined to assisted reproduction but also extend to natural conception. However, it should be noted that by delaying natural conception, prospective parents directly affect the numerical identity of the future individual, and so cannot harm or benefit them. If the timing of natural conception is justified, it is not in virtue of harming or benefiting the future child. In the case of repro‐timing harm and benefit, however, we have strong person‐affecting reasons relating to the wellbeing of the future child, as their identity is unaffected by the delayed implantation. In light of this, repro‐timing harm and benefit can be considered a way of reconciling our intuitions about the need to avoid a bad start in life *for* our future child with the fact that such a decision directly affects *that* specific child.

Some might argue that this kind of harm and benefit, while possible in principle, may have limited application. This may be due to two reasons: first, repro‐timing harms and benefits are only possible once the couple has undergone IVF and the embryos already exist. Second, avoiding repro‐timing harms or securing repro‐timing benefits might generate very demanding obligations. These obligations would require parents to interrupt a parental project—which has a very significant existential and biographical weight— forcing them to suspend and then restart a process that can be emotionally, physically, and financially burdensome. There is no doubt that these considerations are morally relevant in evaluating procreation; thus, some might argue that they could systematically override repro‐timing harms and benefits. In other words, it is doubtful that repro‐timing harms and benefits are morally relevant.

Regarding the first reason, we think it is plausible that more people will use IVF in the future. Indeed, people now tend to want children later in life, and this could increase fertility problems, which might be addressed through assisted reproduction. The growing recognition of LGBTQ+ couples and their desire for parenthood may also contribute to increased IVF uptake. This could significantly extend the contexts in which repro‐timing considerations might apply. The second reason, however, is more complex and requires further analysis, to which the remaining sections of the paper are dedicated.

## 4 Repro‐Timing Benefits and Harms in the Short Term (< 1 Year)

In 4.1, we present findings from the empirical literature on how season and month of birth affect life prospects. This literature provides strong evidence that children born at different points in the year are significantly advantaged or disadvantaged across their lifetimes. We argue that these effects are significant enough that prospective parents engaged in the IVF process have a pro tanto reason to schedule implantation so that their children are born during the more beneficial times of the year. In 4.2, we consider countervailing reasons and argue that these are likely outweighed by the benefits to one's children of delaying implantation.

### 4.1 How Time of Birth Within a Year Affects Wellbeing

There are two main ways in which the time of birth within a year affects the wellbeing of children. The first is called the *Relative Age Effect* (RAE). The RAE is a phenomenon whereby children born earlier within a relevant selection period have different outcomes than those born later within the selection period. The effect of RAE is clearest in education. The academic year in the Northern Hemisphere often has a cut‐off point for enrollment in August or September. If the cut‐off point is the 1st of September, then a child born on the 2nd of September will be almost a full year older than a child born on the 31st of August the following year, yet they will be enrolled in the same cohort. This means that the relatively older child will be significantly more developed than the relatively younger one, thus giving them an early advantage in school. On average, relatively older children do better than relatively younger ones.

This effect is well studied. Dhuey et al. [23] assessed large‐scale population‐level birth and school data from the state of Florida and found evidence for a positive relationship between school starting age and cognitive development from age 6 to 15. A survey of 21 studies on the RAE by Urruticoechea et al. [24] found that relatively younger students (a) obtain significantly lower mean scores in cognitive and motor tests, (b) have a higher repetition rate, and (c) have a lower capacity for socialization. Yamaguchi et al. [25] found that younger students in a grade cohort have lower cognitive and noncognitive skills. While the gap in cognitive skills decreases over time from fourth to ninth grade, the gap in noncognitive skills (conscientiousness, self‐control, self‐efficacy) remains constant. Furthermore, they found that the relatively younger students and their parents make efforts to compensate for their disadvantage by studying more, being more likely to attend a prep school, and spending less time than their older peers on art, sport, and music. They also found that relatively younger students had, on average, poorer relationships with teachers and peers, and were less likely to enter higher‐quality high schools.

If the RAE were restricted to the first years of school and had no significant long‐term effects, then prospective parents engaged in IVF might not have a strong reason to favor certain times of birth over others. There is significant evidence, however, that the RAE confers advantages on the relatively older children that are much longer lasting. Dhuey et al. [23] find that August‐born children are more likely than their September‐born peers to be incarcerated in their teenage years, and they are less likely to attend college, complete college, and are 7.2% less likely to graduate from a selective college. Autumn‐born applicants are 25% more likely to be accepted into an undergraduate program at Oxford or Cambridge than summer‐born applicants [26]. Fukunaga et al [27] found evidence of the RAE among Nobel laureates from the United Kingdom. Those born in the quarter from September to November were significantly overrepresented—41.3% of UK Nobel Laureates were born in this quarter of the year.

The RAE also has significant effects on sporting achievement, again with the relatively older athletes being significantly overrepresented [28, 29, 30]. The RAE has also been shown to have an effect on attaining leadership positions in adulthood. Muller and Page [31] have found that top politicians in the United States are about 50% more likely to be among the oldest in their cohort, and they speculate that this is caused by “human capital acquisition,” that is, the idea that people develop important skills, like leadership, early in life, and these compound over time. Du et al. [32] found that CEOs are significantly less likely to have been born in the summer months, and they attribute this to the relatively older students having developed leadership skills in school (see also [33]).

The evidence is quite clear—relatively older students have an early advantage in school, which can compound over time, resulting in lifelong advantages in educational attainment, sports achievement, and taking on leadership roles. The results seem especially pronounced in highly competitive fields such as elite sports, admission to elite universities, and politics and high‐level business [31]. Being among the oldest in one's school cohort seems so advantageous that prospective parents engaged in the IVF process have a pro tanto reason to try to arrange for their children to be born as early as possible within the selection period. In the Northern hemisphere, this means arranging for one's child to be born in September. Conversely, prospective parents should try to avoid having their children born in August, as these relatively younger children face significant disadvantages, which Crawford et al. [34] have called ‘the August birth penalty’.

The second way in which time of birth within a year affects wellbeing is called the Seasonal Birth Effect (SBE). While the RAE is a social effect, the SBE is a biological phenomenon. Those born in different seasons will face different environmental conditions, seasonal viruses, availability of different foods, and so on. This results in people born in certain seasons being more or less likely than others to contract various diseases and illnesses. For example, those born in winter or spring have a slightly greater chance of developing schizophrenia than those born in summer and autumn [35], while those born between April and June have a 17% greater risk of suicide than those born in autumn to early winter. The data here is more complex, with birth months offering different risk profiles for various health problems [36]. Unlike with the RAE, there is no simple answer to which season is ‘best’ for health outcomes, and prospective parents may need to consider which health risks their own children are genetically predisposed to. There is, however, significant data suggesting that those born in spring and summer have slightly shorter lifespans (around 4 months) on average than those born in autumn and winter [37, 38].

### 4.2 Countervailing Reasons

The most obvious drawback to strategically timing implantation to ensure birth in the optimal season is that prospective parents are no longer free to choose the time of implantation they prefer. This may seem especially unfair as it is a cost which applies only to prospective parents engaged in the IVF process, who already face significant reproductive burdens compared to parents who conceive naturally.^2^ Compared to the benefits that the child will gain by being born at an optimal time, however, this burden seems relatively insignificant, especially since the delay in implantation could be as little as a month. A related objection is that by delaying implantation, parents and child get to spend less time together. Assuming that parental lifespan is unaffected by the delayed implantation, we should expect that a delay of *n* months means that parents and child will get to spend n months fewer together over their lives. Again, since the delay in implantation is relatively short (between 1 and 9 months to ensure birth in the optimal season) and considering the extent of the lifetime benefits to the child of an optimal birth time, it seems to us that the benefits easily outweigh the costs. A third consideration against strategic implantation is the additional financial cost prospective parents must bear. The cost for embryo freezing varies but can be as little as a few hundred dollars in the US and around £700 in the United Kingdom for 1 year.^3^ Compared to the benefits and to the cost of IVF itself, this cost seems trivial.

More significant reasons against strategic implantation might be found in the health effects on the mother and child. If it is the case that giving birth at certain times of the year is associated with worse health outcomes for the mother, then this would give prospective parents a reason not to time implantation to give birth at that time. We could find no research on how the season of giving birth affects maternal health. One possibility is that postpartum depression might be more likely at certain times of the year, at least for some people. For example, people affected by Seasonal Affective Disorder may experience depressive symptoms in Autumn and Winter. If a person affected by SAD gave birth in these months, it might increase the risk of postpartum depression. More research is needed on this question, but this gives a possible countervailing reason against strategically implanting the embryo for an Autumn birth for some people.

As indicated above, different seasons have different risk profiles for various diseases. Though an Autumn birth is advantageous in many ways, it is also associated with a higher risk of some diseases, including respiratory diseases. If prospective parents were to time implantation so that their child is born in Autumn, this might confer some advantages, but it could also result in the child having a respiratory disease they might not have had had they been born in another season. We can imagine that parents might feel responsible if their child develops asthma, for example, after being strategically implanted for a September birth, since their choice to strategically implant the embryo led to the child being born in a month with an increased risk factor for that disease. Could this give prospective parents engaged in the IVF process a reason not to strategically time implantation? One way to argue for this is to say that, even if a September birth is on balance beneficial for the child, parents have a greater reason to avoid causing harm to their child than they do to secure benefits for them, and so they should avoid birth months with greater risk. This approach is unlikely to work, given that every month has its own risks—whichever birth month the parents choose, they will be exposing their child to the risk of harm. Nor can parents simply refrain from choosing a birth month—choosing not to delay implantation is itself a choice for the birth month, 9 months after the fertilization of the embryo. Since a birth month must be chosen, it seems more sensible that the parents choose whichever month is likely to be optimal for the welfare of the future child, also considering any harms and benefits to the parents. If that results in the child being born with a disease which that birth month has a higher risk for this seems like a piece of moral bad luck for which the parents should hardly be considered blameworthy. In choosing under uncertainty, it is often the case that the best we can do is make the choice that has the highest ex ante value. The choice of birth month for prospective parents involved in the IVF process is no different, and they should not be blamed if, against the odds, that choice has some negative effects for their child.

A final consideration which might count against strategic implantation is that it is unfair to confer such advantages on one's future child. This could be because parental partiality in general is difficult to justify.^4^ Alternatively, it could be the case that while parental partiality is permissible or even required, there could be something particularly unfair about the advantages conferred by repro‐timing for optimal birth time. In particular, it might seem that benefitting one's child by taking advantage of the RAE raises issues of fairness that other forms of parental partiality do not, since it benefits one's own child at the expense of other children. It does seem unfair that some children gain significant advantages over others in virtue of time of birth, and several social scientists who have studied RAE have suggested that policymakers ought to try to correct for this advantage. We will not try to answer the question of whether policymakers ought to try to mitigate the effects of the RAE. First, we will simply note that, even if we think that the advantages accruing to relatively older children in virtue of this effect are unjust, this does not entail that parents ought to avoid seeking these advantages for their children. Parents, qua citizens, might have reason, for example, to support educational policies that mitigate the effects of the RAE. However, we take it that as parents their strongest duties regard the welfare of their own children and that they have reason to promote their children's welfare even if this is not perfectly just from the point of view of public policy. Analogously, while parents, qua citizens, might have reasons to support educational reform so that state schools are just as good as private ones, qua parents, they may still have special obligations toward, or prerogatives regarding, their own children, which make it acceptable to send them to a private school.

Secondly, given that the timing of natural conception is identity‐affecting, it is not clear that naturally conceived children are harmed by the parental decision that results in their being born in suboptimal months, whereas those children born through IVF would, in fact, be harmed by being born in these months, as the timing of their births is not identity‐affecting. Naturally conceiving prospective parents may have no duty to ensure their children are born in an optimal month, while those engaged in IVF do, since the very same child would be born regardless of the month of birth and so would be harmed by being born at a less opportune time. If the unfairness of the RAE is about conferring benefits and harms on children, then naturally conceived children are not harmed by the RAE, even if children born by IVF are benefited by it.

## 5 Repro‐Timing Benefits and Harms in the Medium Term (1–5 Years)

In the previous section, we discussed how waiting a few months before implanting the embryo could significantly affect the well‐being of the future individual. However, prospective parents might also encounter moral reasons to delay implantation for several years, as prevailing social and economic conditions and other events could significantly affect the quality of life of their child.

### 5.1 Reasons to Delay Implantation in the Medium Term

Let us assume that a couple that has already undergone IVF to have a child—and therefore created embryos—discovers that war has broken out in their region and is expected to last for years. If the parents decide to proceed with implantation, the adverse conditions may negatively affect the well‐being of their future child. For example, it is possible that food may be rationed, which could lead to malnutrition in children, further exacerbated by lack of access to basic healthcare [46]. This could not only affect the child's development but also increase the likelihood of infectious diseases, as well as mortality. The risk of mortality would further increase when considering not only deaths due to biological factors but also the possibility that the child might die directly due to the fighting. War could also force the family to flee their country, condemning them to live in difficult circumstances and to suffer from family disruption. In general, in line with Bürgin et al. [47], the experiences of children in war and those who flee seem incompatible with the basic needs of children, including the needs for security, shelter, food, parental care, and education [48]. This evidence seems to provide prospective parents a pro tanto moral reason to avoid transferring an embryo into the uterus under these circumstances.

Economic circumstances could also raise the possibility of repro‐timing harm and benefit. Imagine a couple is undergoing IVF, and subsequently, one of the partners loses their job. Additionally, let us assume that the job loss occurs within a broader economic crisis, making it uncertain whether the partner will be able to find another job with comparable pay in the near term. The lack of economic resources could create a situation where the child is worse off compared to a scenario in which the parents wait a few years before transferring the embryo into the uterus. This situation could not only prevent the child from accessing a higher level of healthcare or a better quality of life in general, but it could also negatively affect their emotional and cognitive development due to the high stress experienced by families in economic crises.

A similar effect could occur even in situations where the familial problem is not financial but affective. For example, if a couple who decides to have a second child through IVF discovers that their first child has an illness that requires significant parental involvement for some years, procreating under these circumstances could lead to serious issues for the future child. The limited attention the future child is expected to receive in the first years of life could have a significant impact on their well‐being and opportunities. The early years of life are a crucial period for brain development: by the age of three, a child's brain has already reached about 90% of its adult size [49]. Evidence has shown that the interactions a child has with caregivers are essential for the initial development of brain connections, as they facilitate the creation of a vast number of neural connections [50, 51, 52, 53]. This shows that a poor emotional bond can have implications for the risk of developing depression, anxiety disorders, or learning and memory deficits [53].

The cases described above can be defined as repro‐timing harms that depend on external conditions, such as the outbreak of war, job loss in the context of an economic crisis, or the emergence of family problems that compromise the parents' emotional resources. Nonetheless, there is at least another case worth mentioning in which the repro‐timing harm parents might cause is determined by a health condition of the future individual that they could have avoided by delaying implantation. Suppose that parents undergoing IVF discover through PGD that the only viable embryo for transfer has a condition that significantly impairs the well‐being of the future individual. However, they learn that the European Medical Agency is approving a drug that, if administered in the first days of the unborn child's life, would completely reverse the effects of the disease. The doctors inform the parents that, although very promising, the drug will not be available for at least another 2 years. If the parents decide to implant anyway, the child would be born with a disease that could have been avoided if the transfer had been postponed by 2 years, thus incurring a repro‐timing harm. This could give the parents moral reasons to delay the pregnancy.

Whereas the aforementioned cases primarily present instances of avoiding repro‐timing harm because the circumstances of birth can significantly compromise the future individual's well‐being, it is also possible to imagine cases where parents might have reasons to delay the pregnancy because this could promote greater well‐being for the future child. Prospective parents undergoing IVF who have a reasonable expectation of increasing their income or improving their social situation in the coming years might have moral reasons to delay the pregnancy to benefit the child, ensuring greater access to educational, healthcare, and social facilities and other assets for promoting their well‐being.

### 5.2 Countervailing Reasons

Reasons to delay implantation for several years should be balanced with other considerations, including some non‐negligible factors that parents encounter when they face the possibility of repro‐timing harm or benefit for the future individual. We must acknowledge that waiting several years to reproduce may be very demanding. It means putting a parental project on hold—something that carries deep personal significance for those who decide to reproduce via IVF and who have a strong desire to become parents. This is even more salient when we remember that undergoing IVF is often associated with physical and psychological burdens: those who experience unsuccessful reproductive cycles frequently face increased anxiety and worries about their ability to conceive [54]. This emotional strain adds to the significant stress already associated with infertility, which often motivates couples or individuals to seek out IVF [55].

Another factor to consider is that delaying implantation by several years would also result in parents being slightly older than they would have been if they hadn't postponed the pregnancy. This could affect the future child's quality of life in at least two ways: first, older parents may experience greater parental stress, given that raising a child is very demanding, and, over time, they might lose the necessary energy. This could provide reasons to transfer the embryo as soon as possible to avoid any potential impact on the child's well‐being and development. Qualitative studies by Jadva and colleagues [56] and Mac Dougall et al. [57] found that some parents felt “tired and slow” due to their age; second, it could be argued that postponing pregnancy and, therefore, parenthood might ultimately be negative, as it would mean the child spends less time with their parents. As for the first consideration, a recent literature review seems to reject this claim: although older parents feel the social stigma for their age, this does not seem to affect the child‐parent relationship, even though the evidence on child adjustment is inconclusive [58]. The second reason must be taken more seriously, especially considering that, albeit limited, evidence on this certifies that mothers over the age of 40 in qualitative studies were indeed concerned about having less time to dedicate to their children, or that mothers also reported their children as being ‘preoccupied’ with their parents' mortality as a result of their older age [59]. However, it is important to highlight that there is a conceptual and experiential difference between qualitative and quantitative. Therefore, while it is true that procreating later may lead to less time spent with one's parents, greater economic and emotional stability could ensure qualitatively better time with their children.

Generally speaking, the countervailing reasons we encountered in this section prompt us to be cautious regarding the overall strength of the moral reasons to repro‐timing benefit future individuals in these circumstances, as these reasons should be carefully balanced with other morally relevant considerations. However, it is worth noting that our argument is compatible with a certain asymmetry between avoiding repro‐timing harm and conferring repro‐timing benefit. Specifically, the moral reasons to avoid repro‐timing harm may be stronger than those for conferring a slight improvement in the quality of life to a person whose life would already be sufficiently good. Consequently, when balancing these considerations to make an *all‐things‐considered* decision, the pro tanto reasons to confer repro‐timing benefits may carry less weight than the reasons to avoid repro‐timing harm.

## 6 Repro‐Timing Benefits and Harms in the Long Term

So far, we have restricted our attention to the short and medium term, that is to delaying implantation by a few months or years. However, embryos can remain frozen for much longer than this without any ill effect on the resulting children. As mentioned in the first section, children have been born from “adopted” embryos that have been frozen for several decades, and some experts claim that embryos can be safely preserved indefinitely.^5^ It is technically possible then for prospective parents (or in this case perhaps simply “reproducers”) to arrange for their embryo to be implanted by someone else decades or more in the future. This raises the question of whether reproducers engaged in the IVF process could repro‐timing benefit their future progeny by making such arrangements, and whether they might have reason to do so. In this section, we will briefly investigate this question.

The historical era one is born into can have large effects on one's wellbeing. Given the economic, scientific, medical, moral, and cultural developments of the last 100 years, it is plausible that people born at the end of the 20th century have better lifetime wellbeing levels than people born at the end of the 19th. Since that progress shows no obvious signs of stopping, it is reasonable to think that people born at the end of the 21st century will likewise have better lifetime wellbeing levels than people born at the end of the 20th.^6^ The same rationale that gives prospective parents a pro tanto reason to strategically delay implantation to provide repro‐timing benefits to their future children in the short and medium term also applies over longer time‐scales. There is no technical reason why longer delays would be impossible, since embryos can be safely frozen indefinitely. Nor is it plausible that prospective parents' moral reasons to benefit their children are only operative over the short and medium term. Nor are far future benefits to wellbeing worth less than near future ones – almost all philosophers who have considered it reject the idea that we should embrace pure time preference to discount the value of future benefits.^7^ If we think that this historical progress will continue and that the future will be better than the present, this gives a pro tanto reason to arrange for embryo implantation in the future to provide greater repro‐timing benefits to one's child.

Furthermore, the pace of progress might increase significantly. It is a common thought among futurologists and longtermists that the future may be dramatically better than the present, with humans living lives that are many times better than anything we can imagine today. Ideas like these appear in Parfit [3], Pearce [67], Kurzweil [68], Bostrom [69], Ord [63], and MacAskill [66]. Advances in Artificial Intelligence, genetic engineering, and medicine might allow humans in the future to live indefinitely long lives with superhuman intelligence, enjoying degrees and kinds of positive welfare beyond anything that is possible, or even conceivable, today. If these utopian dreams come true, then it is in principle possible that the repro‐timing benefits prospective parents could confer on their future children by arranging for implantation in the far future may be orders of magnitude greater than the benefits they can secure by strategically timing implantation in the short or medium term.

There are, of course, many significant costs and difficulties with such long‐term delays to implantation. Most obviously, the prospective parents might not have any relationship with their future child, who could be implanted and born long after her parents have died. This would be a massive sacrifice on the part of the parents who would be giving up on having a loving relationship with their child in the hopes of securing a much better life for them. Parents already make such sacrifices for their children—for example, parents sometimes give their children up for adoption in the belief that they will have a better life with adoptive parents who are in a better position to care for them. We do not think badly of such people—rather, we think that they have made a difficult decision that is motivated by doing the best they can for their child. Prospective parents who give their child up for “intertemporal adoption” by arranging for implantation in the far future might have similar motivations and might find the sacrifice worth it as a means to securing the best possible life for their child. There are important differences between the cases too, however. For example, we might think that a parent who gives her child up for adoption because she is not capable of caring for it is motivated by the idea that she is not able to give the child a good enough life, not by the desire to give the child the best life possible. Prospective parents engaged in the IVF process presumably are generally capable of giving their children good enough lives. By arranging for implantation in the far future, they would be motivated by maximizing the benefits to their child, rather than ensuring that the child has a good enough life, which they can already provide.

Another difference concerns the practical difficulties of arranging for the implantation of the embryo in the far future rather than simply delaying implantation for a few months or years. In the short and medium term, prospective parents can be reasonably confident that they will be able and willing to implant the embryo themselves. In the long‐term future case, it is less clear how prospective parents could ensure that their embryo is implanted. As mentioned above, there are fertility clinics (generally Christian ones) in which couples can “adopt” surplus embryos from other couples' IVF treatments. It might be possible to make arrangements with such a clinic to have their embryo implanted in an adoptive mother in the future. Alternatively, it might be possible to make arrangements with the clinic in which the embryo was frozen so that the embryo is kept frozen for a certain amount of time before an adoptive couple is sought for implantation.

Finally, one might worry that the far future could in fact turn out to be worse than the present, and so delayed implantation would actually be a repro‐timing harm. However, it should be possible for the prospective parents to stipulate certain conditions that must be met if the embryo is to be implanted at all. If the future is less good than the present, then the embryo need not be implanted. This would not be a harm, since not being caused to exist can be neither better nor worse than being caused to exist.

In this section, we have argued that the same rationale, which gives prospective parents a pro tanto reason to delay implantation in the short and medium term, is also applicable in the long term. If the far future is likely to be much better than the present or near future, then parents may have pro tanto reason to try to arrange for their embryos to be implanted in the far future, to provide the greatest repro‐timing benefits possible to their children. We do not claim that they have an all‐things‐considered reason to do so, especially since long‐term future implantations are accompanied by extreme sacrifice on the part of the parents, as well as very significant costs and difficulties not found in the short‐ and medium‐term cases.

## 7 Conclusion

In this paper, we argued for the existence of a novel counterfactual comparative harm or benefit that arises in assisted reproduction in light of the availability of IVF and embryo cryopreservation: repro‐timing harm and benefit. Recognizing this could lead to the emergence of person‐affecting reasons for procreators within the IVF process to delay the timing of the designated embryo's transfer into the uterus. We then explored potential real‐world applications of this concept, presenting moral reasons that emerge in short‐, medium‐, and long‐term scenarios. We believe that exploring these applications could help inform and guide the decisions of prospective parents accessing assisted reproduction. Notwithstanding, we did not claim that parents have an all‐things‐considered reason to delay implantation, but just pro tanto ones; reasons emerging from repro‐timing harm and benefit would need to be balanced with other morally relevant countervailing reasons in the context of procreation. Further research is needed to identify appropriate ways to balance all morally relevant considerations emerging from reproduction, especially when the interests of the parents and the well‐being of the future children, or society more broadly, conflict. The aim of this paper was more modest: to demonstrate that repro‐timing harms and benefits are possible and that the concept has real‐world applications.

## Data Availability

Data sharing is not applicable to this article as no new data were created or analyzed in this study.

## Endnotes

1 Notice that the approaches to procreative ethics discussed in this section do not exhaust the debate on the procreative duties. For instance, other approaches focus on procreative motivations and intentions [12, 13].

2 One of the authors has already argued at length that developments in ART are accompanied by new moral duties [11, 13, 39].

3 See Reprotech [40], Pinnacle Fertility [41], Cambridge IVF [42], and IVF London [43].

4 See Douglas [44, 45].

5 On the general practice of ‘adopting’ frozen embryos, see Taylor‐Coleman [59]. On the long‐term viability of frozen embryos, see Saplakoglu [60] and Reprotech [61].

6 For an optimistic account of humanity's historical progress so far, see Pinker [62]. For speculations on how that progress could continue, see Ord [63].

7 See Cowen and Parfit [64]; Ord [63]; Greaves and MacAskill [65]; MacAskill [66].
